# A multimodal tactile dataset for dynamic texture classification

**DOI:** 10.1016/j.dib.2023.109590

**Published:** 2023-09-16

**Authors:** Bruno Monteiro Rocha Lima, Venkata Naga Sai Siddhartha Danyamraju, Thiago Eustaquio Alves de Oliveira, Vinicius Prado da Fonseca

**Affiliations:** a School of Electrical Engineering and Computer Science, University of Ottawa, Ottawa, ON, Canada; b Department of Computer Science, Lakehead University, Thunder Bay, ON, Canada; c Department of Computer Science, Memorial University of Newfoundland, St. John's, NL, Canada

**Keywords:** Texture classification, Dynamic exploration, Tactile sensor, Machine learning

## Abstract

Reproducing human-like dexterous manipulation in robots requires identifying objects and textures. In unstructured settings, robots equipped with tactile sensors may detect textures by using touch-related characteristics. An extensive dataset of the physical interaction between a tactile-enable robotic probe is required to investigate and develop methods for categorizing textures. Therefore, this motivates us to compose a dataset from the signals of a bioinspired multimodal tactile sensing module while a robotic probe brings the module to dynamically contact 12 tactile textures under three exploratory velocities. This dataset contains pressure, acceleration, angular rate, and magnetic field variation signals from sensors embedded in the compliant structure of the sensing module. The pressure signals were sampled at 350 Hz, while the signals of the other sensors were sampled at 1500 Hz. Each texture was explored 100 times for each exploratory velocity, and each exploratory episode consisted of a sliding motion in the x and y directions tangential to the surface where the texture is placed. The total number of exploratory episodes in the dataset is 3600. The tactile texture dataset can be used for any project in the area of object recognition and robotic manipulation, and it is especially well suited for tactile texture reconstruction and recognition tasks. The dataset can also be used to study anisotropic textures and how robotic tactile exploration has to consider sliding motion directions.

Specifications Table

**Table:** 

Subject	Robotics
Specific subject area	Tactile texture sensing, perception, and recognition.
Type of data	Python pickle file, Python scripts for parsing and plotting
How the data were acquired	The tactile data is captured using a tactile-enabled finger holding the sensing module mounted on a plastic base. The fingertip round shape allows for a more efficient 2D texture exploration. The module provides data from a nine degrees-of-freedom Magnetic, Angular Rate, and Gravity (MARG) system and deep pressure data from a base-mounted barometer encased in polyurethane rubber. This sensor placement provides the ability to capture the module's deformation. A sensing module holder was designed to simulate human natural exploration using the index finger model. An XY-recorder is used to hold each tactile texture. During each exploratory episode, the recorder moved the textures along the XY plane while the tactile finger stayed in a fixed position above the textures and was raised and lowered between episodes to bring the sensing module to contact the textures. More information about the data acquisition setup can be found in [1]. More information on the design of the sensing module utilized in the data acquisition can be found in [2].
Data format	Raw
Description of data collection	Data collection was conducted under specific conditions to study the recognition of tactile textures using a tactile-enabled finger with a multi-modal sensing module. The data points were generated by exploring 12 different textures, including plastic pencil cases, plastic placemats, cork, denim fabric, felt, polyester cloth, and cotton scarf. Inclusion criteria involved selecting textures commonly encountered in everyday environments. The data collection process involved linear and 2D exploration tasks, where the finger remained fixed while the textures moved in predefined directions. The data points were collected at three exploratory velocities: 30 mm/s, 35 mm/s, and 40 mm/s. Overall, the data collection focused on capturing the sensory responses of the tactile sensing module to different textures and exploratory movements, providing a comprehensive dataset for further analysis and research in the field of texture recognition and robotic manipulation.
Data source location	*Institution: Memorial University of Newfoundland,* *City/Town/Region: St. John's, NL* *Country: Canada* *Institution: University of Ottawa,* *City/Town/Region: Ottawa, ON* *Country: Canada*
*Data accessibility*	Repository name: Multimodal Tactile Texture Dataset Data identification number: 10.17632/n666tk4mw9.1 Direct URL to data: https://data.mendeley.com/datasets/n666tk4mw9/1
*Related research article*	*B. M. Rocha Lima, T. E. Alves De Oliveira, and V. P. Da Fonseca, “Classification of Textures using a Tactile-Enabled Finger in Dynamic Exploration Tasks,” in 2021 IEEE Sensors, Sydney, Australia: IEEE, Oct. 2021, pp. 1–4. doi:*10.1109/SENSORS47087.2021.9639755.

## 1 Value of the Data

• This dataset is valuable for advancing the development of robotic tactile sensors, enabling robots to interact through touch with objects. These interactions can be static, usually generating tactile images [3,4], or dynamic, generating signals that vary over time [5], [6], [7], [8]. Our dataset focuses on dynamic tactile data for texture characterization but utilizes a set of commonly used tactile textures instead of synthetic gratings [9] or macroscopic profiles [10].
• This dataset is also valuable because it aims to avoid a halt in the research of tactile perception of textures, considering that previous research, such as [11,12], have not maintained the availability of their data. In [13], two datasets for the investigation of visuo-tactile data representation are proposed but have yet to be made available to the public at the moment. The dataset proposed here complements the proposed [13] by making valuable data from a different set of sensors open to the public at https://data.mendeley.com/datasets/n666tk4mw9/1.
• Researchers in the field of robotics can benefit from these data, utilizing them to explore and enhance texture recognition and manipulation capabilities. This dataset may also expose researchers from other areas to multimodal tactile perception. For instance, the same data was used for heart rate detection in the biomedical engineering field [14], haptic surface reconstruction [15], and tactile object recognition [16], which demonstrates the versatility of this data and its possible use in other tasks.
• In [17], the authors explore spike depiction for tactile texture recognition from simulated taxel data using neural networks but have yet to make the dataset available. The authors of [18] present an interesting dataset for texture recognition using data from an accelerometer and a microphone but only explore the 12 textures in one direction. The dataset proposed here complements their work by exploring 2D textures with multiple sensing modalities
• These data can be used to develop and refine machine learning algorithms, leading to more accurate and efficient texture classification in robotic tactile sensing systems. The dataset allows for further insights into the influence of exploratory velocities on texture classification, aiding in optimizing robotic manipulation tasks. For instance, this data was already used in [1] and [19] to classify textures in two different exploratory situations.
• Researchers can also leverage this dataset as a benchmark to evaluate the performance of different machine learning models on sequential tactile data, driving advancements in tactile texture classification.

## 2 Objective

The objective of generating this dataset is to provide a comprehensive collection of data that explores the recognition of tactile textures in dynamic exploration scenarios. The dataset was generated using a tactile-enabled finger with a multi-modal tactile sensing module. By incorporating data from pressure, Magnetic field, Angular Rate, Gravity (MARG) sensors, the dataset aims to facilitate research on machine learning methods for texture classification.

The accompanying dataset can also be used to answer research questions related to the preemptive classification of tactile texture classification, such as few-shot, zero-shot, and sequential learning. The dataset provided will also increase the availability of MARG data for tactile-texture tasks and help develop machine-learning models for such tasks.

It is worth noting that tactile texture classification from sliding motions is an important precursor subtask when a robot explores a surface with the objective of placing an object or modifying such surface. Texture may also be complementary to object identification prior to or under grasp. Two or more objects may have the same global shape, stiffness, and weight but different textures and other microscopic structures.

## 3 Data Description

The dataset contains the raw sensor measurements in data frames stored in pickle files. The dataset also includes a sample script that reads pickle files and uses Python's Pandas library to access their data frame. The data files are organized in a specific folder structure and contain multiple readings for each texture and exploratory velocity. The dataset contains raw data recorded from tactile measurements for different textures and exploratory velocities stored in pickle files. Table 1 shows the dataset folder and subfolder descriptions.

**Table 1:** Sample contractions and their possible expanded forms from the contractions dictionary.

Folder Name	Description
pickles_30	Folder containing tactile data at an exploratory velocity of 30 mm/s.
pickles_40	Folder containing tactile data at an exploratory velocity of 40 mm/s.
pickles_45	Folder containing tactile data at an exploratory velocity of 45 mm/s.
texture_01 to texture_12	Subfolders containing exploratory episodes with barometer and MARG folders for each texture, labeled as texture_01, texture_02, etc.
full_baro	Subfolder containing pickle files with barometer data for each texture.
full_imu	Subfolder containing pickle files with Magnetic, Angular Rate, and Gravity (MARG) data for each texture.

The folder structure for the episodes with an exploratory velocity of 30 mm/s has the following hierarchical structure:

• *Folder*: pickles_30
  ○ *Subfolder*: texture01 to texture_12
    ■ *Subfolder*: full_baro
      • *Pickle file*: baro_<texture_number><episode_number>
    ■ *Subfolder*: full_imu
      • *Pickle file*: imu_<texture_number><episode_number>

The folders for the exploratory episodes with velocities of 40mm/s and 45mm/s follow the same hierarchical structure. There are 12 folders of textures for each velocity, with 100 exploratory episodes in each one. The data is divided into barometer and imu pickle files for each texture of the exploratory episodes. The pickle file name for each episode also indicates which texture and episode it stores. For instance, the pickle file “imu_0101.pkl” indicates the MARG data from texture 01 during exploratory episode 01. In a similar way, “baro_0101.pkl” indicates the MARG data from texture 01 during exploratory episode 01. Table 2 and Table 3 show five consecutive barometer and MARG sensor readings, respectively.

**Table 2:** Sample of five consecutive sensor readings from the pickle file baro_0101. The *baro* column contains pressure measurements from the analog-to-digital converter readings for the barometer sensors.

*Timestamp*	*baro*
00:00:00.314306	185.0
00:00:00.314612	185.0
00:00:00.314915	191.0
00:00:00.315227	191.0
00:00:00.315539	191.0

**Table 3:** Sample of five consecutive sensor readings from the pickle file imu_0101. The *imu_ax, imu_ay,* and *imu_az* columns display the accelerometer readings in *mm/s^2^*. The *imu_gx, imu_gy,* and *imu_gz* show the angular velocity measurements in *rad/s*. The *imu_mx, imu_my,* and *imu_mz* columns show the measurements of the amplitude of the magnetic field surrounding the sensor in the x, y, and z directions of the sensor reference frame. These measurements are in *gauss*.

*Timestamp*	*imu_ax*	*imu_ay*	*imu_az*	*imu_gx*	*imu_gy*	*imu_gz*	*imu_mx*	*imu_my*	*imu_mz*
00:00:00.000000	-0.914585	-2.506131	9.329007	-0.009526	-0.005611	-0.005481	-0.186523	-0.511108	1.902588
00:00:00.000144	-0.914585	-2.506131	9.329007	-0.009526	-0.005611	-0.005481	-0.186523	-0.511108	1.902588
00:00:00.000501	-0.865504	-2.656965	9.321824	-0.009526	-0.005611	-0.005481	-0.176514	-0.541870	1.901123
00:00:00.000817	-0.865504	-2.656965	9.321824	-0.009526	-0.005611	-0.005481	-0.176514	-0.541870	1.901123
00:00:00.001120	-0.865504	-2.656965	9.321824	-0.00952	-0.005611	-0.005481	-0.176514	-0.541870	1.901123

## 4 Experimental Design, Materials and Methods

The experimental design employed a tactile-enabled finger equipped with a specialized sensing module. This finger held the sensing module, which was affixed to a plastic base, creating an arrangement conducive to tactile exploration. We employed a modified XY-recorder setup featuring a pen carrier to enable controlled movements during the data collection. This setup accommodated the labeled textures mounted on a medium-density fibreboard (MDF) base. The exploration of textures took place in a 2D technique, with predefined movements occurring within the XY plane, and to offer a comprehensive solution, it incorporates multiple exploratory velocities (30 mm/s, 35 mm/s, and 40 mm/s).

**Fig. 1:**
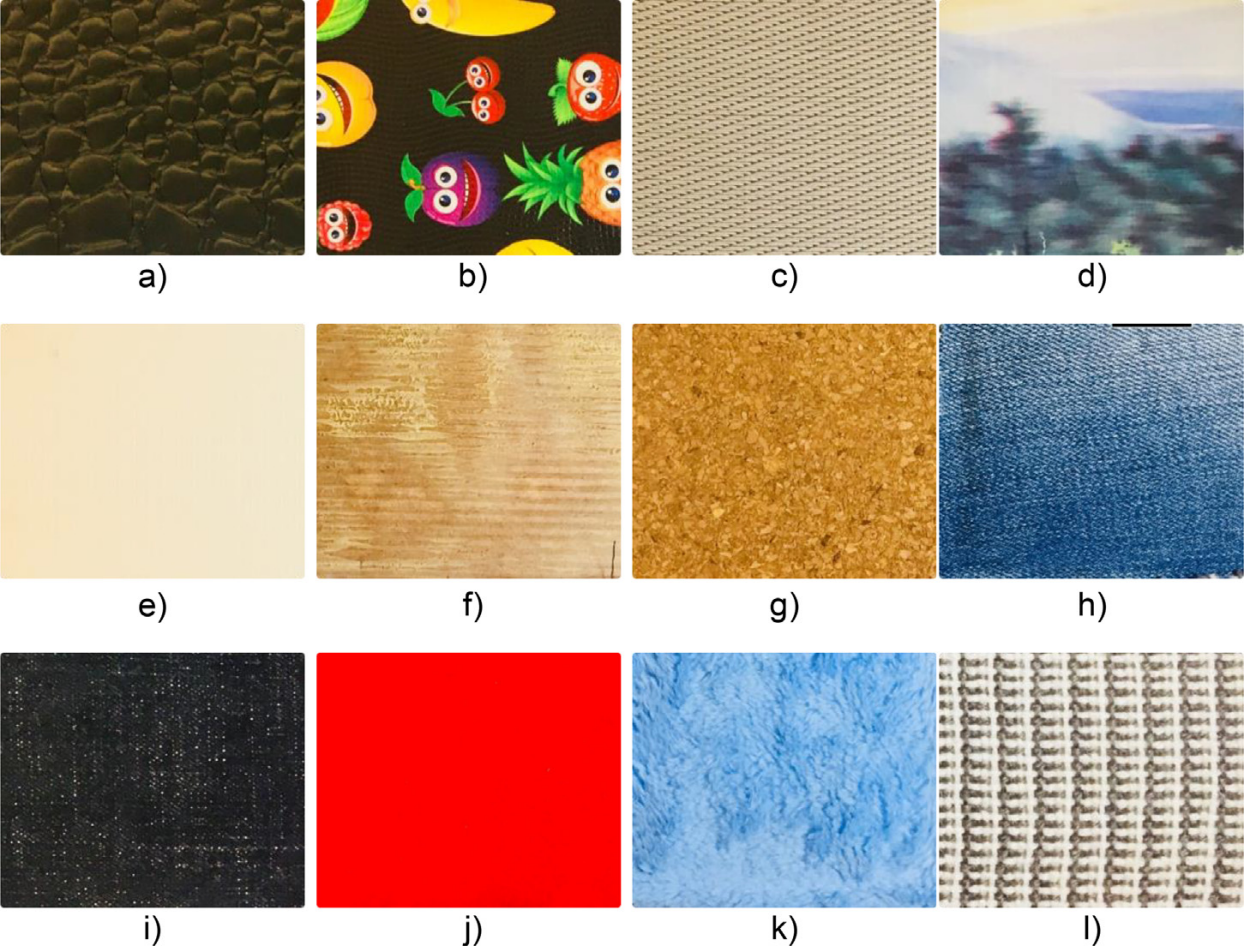
The 12 textures used in the experiments. a) plastic pencil case 1; b) plastic pencil case 2; c) plastic placemat 1; d) plastic placemat 2; e) plastic placemat 3; f) plastic placemat 4; g) cork mat; h) denim fabric 1; i) denim fabric 2; j) felt material; k) polyester cloth; l) cotton scarf.

The materials employed in this experimental setup included the tactile-enabled finger outfitted with a sensing module, a plastic base designed for mounting the sensing module, and an XY-recorder featuring a pen carrier. The textures subjected to exploration consisted of diverse items such as plastic pencil cases, plastic placemats, cork, denim fabric, felt, polyester cloth, and cotton scarf. These textures were securely mounted on MDF bases to ensure stability and consistency throughout experimentation. Fig. 1 shows the 12 tactile textures explored by the robotic finger.

Textures a, b, and g have irregular bumps and are hard materials. Texture c is a high-frequency textured plastic. Texture d has different textures in different directions. Textures e and f have uniform bumps. Textures h, i, j, k, and l are fabric materials, whereas textures j and k are the smoothest among them.

**Fig. 2:**
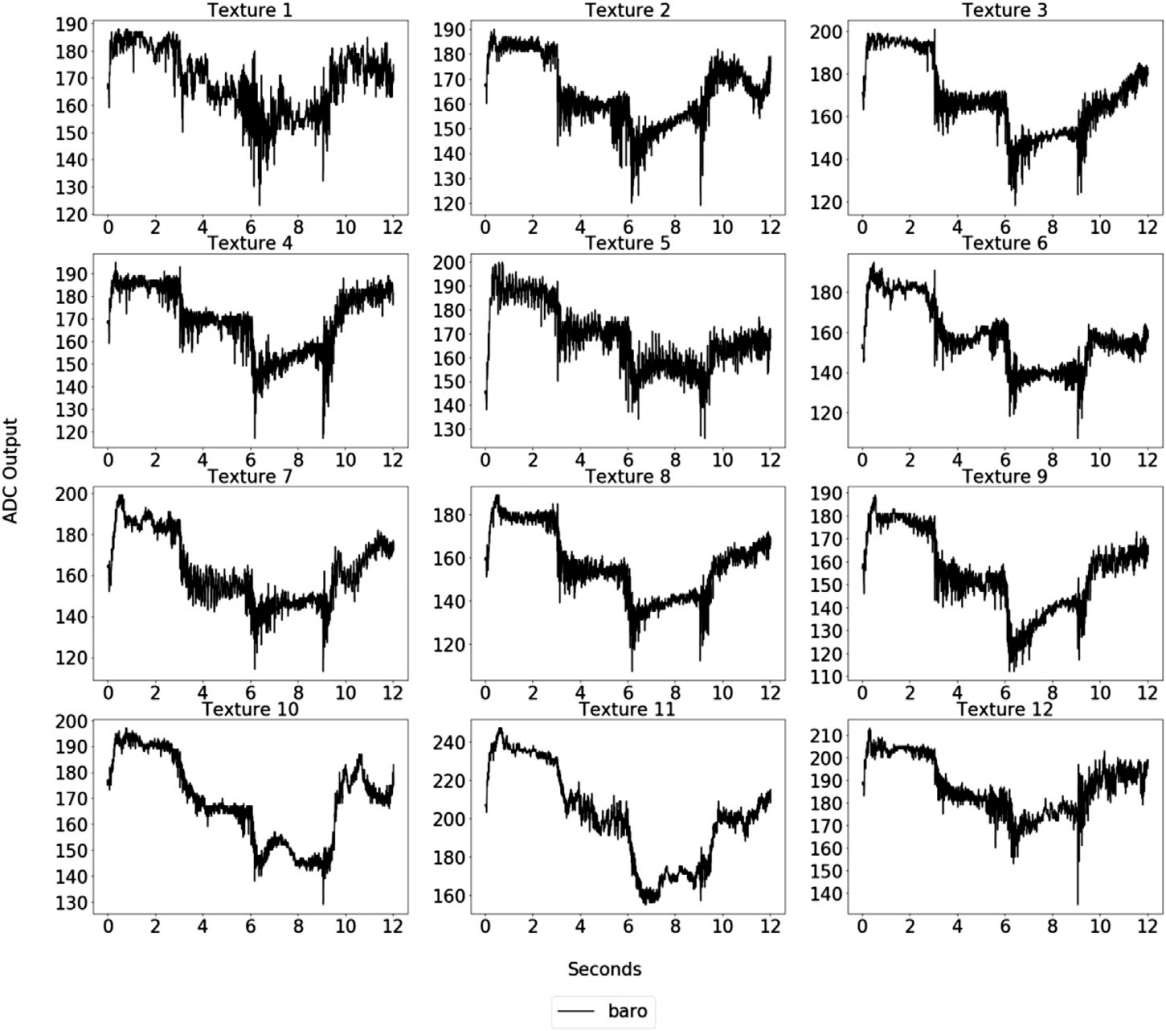
Sample of the barometer data for each texture explored under 30mm/s.

**Fig. 3:**
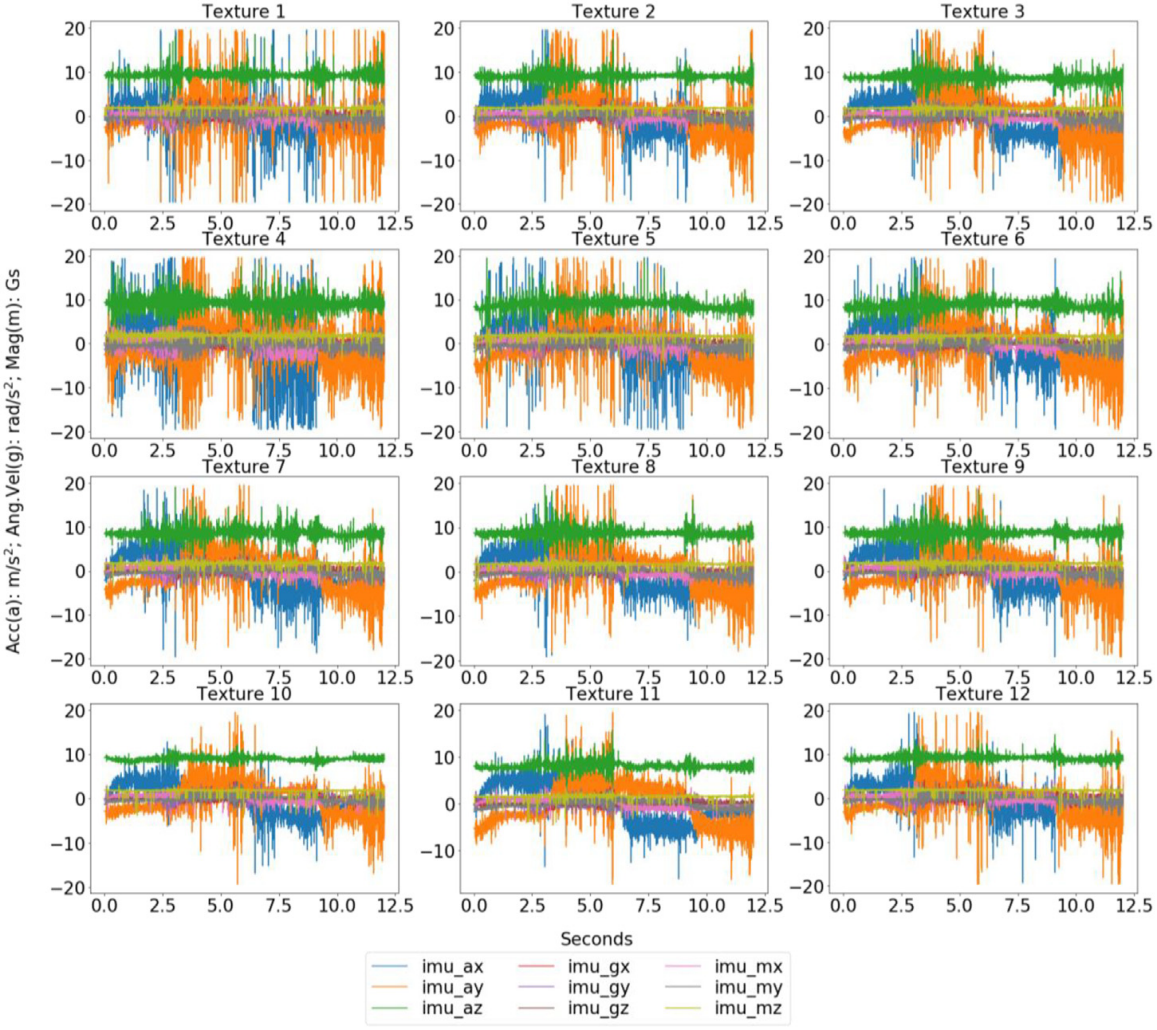
Sample of the MARG data for each texture explored under 30mm/s.

In the execution of the experiment, the tactile-enabled finger remained stationary. At the same time, the textures were moved underneath it, following the predetermined movements outlined within the XY plane. A microcontroller controls the XY-recorder's velocity and position, thus orchestrating the coordinated exploration of the textures. Throughout the exploration, the sensing module captured a range of data, including measurements related to pressure, gravity, angular rate, and magnetic fields. The presence of a barometer allowed for recording deep-pressure data, enabling the observation of module deformation. The data collection process was executed with a combination of hardware components, including the tactile-enabled finger, XY-recorder, microcontroller, and relevant software, ensuring a comprehensive and systematic approach to gathering information. Figs. 2 and 3 show the data collected by the pressure sensor and MARG system while the robotic finger explores the textures at a velocity of 30mm/s, respectively.

## Ethics Statement

This research involving the tactile-enabled finger utilized a tactile sensing module. The study follows ethical guidelines outlined in the Guide for Authors. No human subjects were involved in this study. Therefore, informed consent and ethical committee approval were not required. Animal experiments were not conducted. Data collected from social media platforms was not used in this research. Therefore, participant consent and data redistribution policies were not applicable. The study focused on the technical aspects of the sensor module and did not involve any potential ethical concerns regarding human subjects, animal experiments, or social media data.

## CRediT Author Statement

**Bruno Monteiro Rocha Lima**: Data collection, Data curation, Visualisation, Investigation, and Software. **V Naga Sai Siddhartha Danyamraju**: Writing- Original draft preparation and Software. **Thiago Eustaquio Alves de Oliveira**, Supervision, Conceptualization, Writing- Reviewing and Editing, Validation, and Funding acquisition. **Vinicius Prado da Fonseca:** Supervision, Conceptualization, Writing- Reviewing and Editing, Validation, and Data curation.

## Data Availability

Multimodal Tactile Texture Dataset (Original data) (Mendeley Data)
